# Can Locus of Control Compensate for Socioeconomic Adversity in the Transition from School to Work?

**DOI:** 10.1007/s10964-017-0720-6

**Published:** 2017-07-28

**Authors:** Terry Ng-Knight, Ingrid Schoon

**Affiliations:** Department of Social Sciences, UCL Institute of Education, 20 Bedford Way, London, WC1H 0AL UK

**Keywords:** Agency, Locus of control, SES, Transition from school to work, NEET, Resilience

## Abstract

Internal locus of control is associated with academic success and indicators of wellbeing in youth. There is however less understanding regarding the role of locus of control in shaping the transition from school to work beyond the more widely studied predictors of socioeconomic background and academic attainment. Guided by a socio-ecological model of agency, the current study examines to which extent internal locus of control, understood as an indicator of individual agency, can compensate for a lack of socioeconomic resources by moderating the association between parental disadvantage and difficulties in the transition from school to work. We draw on data collected from a longitudinal nationally representative cohort of 15,770 English youth (48% female) born in 1989/90, following their lives from age 14 to 20. The results suggest that the influence of agency is limited to situations where socioeconomic risk is not overpowering. While internal locus of control may help to compensate for background disadvantage regarding avoidance of economic inactivity and unemployment to some extent, it does not provide protection against long-term inactivity, i.e. more than 6 months spent not in education, employment or training.

## Introduction

The transition from secondary school to work or further education is an important developmental task for youth and ranks high in terms of complexity and relevance for later life outcomes (Buchmann and Kriesi 2011; Schulenberg and Schoon 2012). Youth have to mobilize and take advantage of the opportunities and resources available to them, yet persisting social inequalities continue to shape the challenges they are facing. For example, youth from less privileged family backgrounds are at a greater risk than their more privileged peers of encountering difficulties in finding and sustaining steady and gainful employment during this period (Furstenberg 2008; Lui et al. 2014; Schoon and Lyons-Amos 2017, 2016). There is, however, also evidence to suggest that some youth succeed against the odds, and are able to establish themselves in the labor market or pursue an academic career despite the experience of parental socioeconomic hardship (Duckworth and Schoon 2012; Heckhausen and Chang 2009).

Within this context, individual differences in so-called non-cognitive factors have gained widespread attention in recent years (Heckman and Kautz 2012; OECD 2015), largely due to their ability to predict a range of important outcomes in the adult years, including educational and occupational attainment as well as health and wellbeing independently of parental social background or cognitive ability. Indeed, it has been argued that personality can to some extent compensate for socioeconomic disadvantage (“resource substitution”). However recent evidence shows that high levels of generally valued personality traits such as extraversion and conscientiousness only offer partial compensation for the disadvantage associated with parental socioeconomic status (SES), and that they are by no means sufficient to lead to full catch-up effects (Damian et al. 2015; Shanahan et al. 2014). This suggests that individual characteristics that predict positive outcomes may not necessarily be the same as those that moderate socioeconomic risk processes at the population level. Such an assumption aligns with the concept of “resilience”, which emphasizes a focus on factors that have substantial positive effects in the presence of adversity but often have little or no effects in the general population of low-risk individuals (Rutter 2015). Therefore, more work is required to identify characteristics that reduce risk for disadvantaged youth. The current study shifts focus beyond dispositional traits such as the “big five”, to the more contextualized adaptations that characterize individuals’ attempts to operate as self-determining or agentic beings in a social world (McAdams and Pals 2006). In particular, we focus on the role of internal locus of control as a potential resource factor, enabling youth from disadvantaged background to beat the odds.

Locus of control refers to an individual’s perception of their ability to exercise control over their environment (Rotter 1966) and emphasizes that the choices people make are dependent on expectations that their behavior will result in the desired outcome. For example, “internals” (people high on internal locus of control) believe they have control over their environment and that they can determine what happens in their life, whereas “externals” (people high on external locus of control) believe their lives are dictated by external factors beyond their control. Locus of control maps onto the agentic property of “self-reflectiveness,” which involves the evaluation of one’s personal efficacy and was described by Bandura as the “most distinctly core property of human agency” (2006, p. 165).

The article adds to current debates in three ways. First, we adopt a multi-dimensional approach in conceptualizing SES and unpack the effects of different dimensions of SES on internal locus of control and transition experiences. Second, we examine the role of internal locus of control in shaping the transition from school to work in addition and beyond the influence of SES and academic attainment. Third, we assesses interactions between SES and internal locus of control to assess whether high levels of agency may overcome specific aspects of disadvantage and test whether agency predicts post-school transitions differently for advantaged and disadvantaged youth.

## A Socio-Ecological Model of Agency

The study is guided by a socio-ecological model of agency (Schoon and Lyons-Amos 2017) testing the interplay of parental SES and internal locus of control in shaping the school-to-work transition in a prospective longitudinal design using a population representative cohort of youth. The model is informed by sociological life course theory (Elder and Shanahan 2006) with its emphasis on multiple sources of influence on individual lives, social cognitive theories of human action (Bandura 2006) and Eccles et al.’s (1993) person-environment fit theory. The model was introduced to investigate how objective socioeconomic conditions affect individual thinking, feeling and behavior, and to examine the extent to which youth are able to steer the courses of their lives despite constraining forces of social structure. Individual agency is understood to be shaped by opportunity structures, social networks and institutions, taking into account the impact of multiple socioeconomic risk factors that influence everyday experiences.

In this study, we test two assumptions of this model in the context of post-school transitions: socialization and interaction effects. First, we assess whether there are direct and potentially corrosive effects of low SES on the expression of agency (socialization effects). Second, we examine interaction effects between SES and agency regarding the experiences of youth after completing compulsory schooling, focusing on the amount of time spent not in education, employment or training (NEET). In particular, we test a) if there is an interaction between SES and agency in shaping transition experiences; b) if there are compensatory effects (i.e. “resource substitution” or “resilience” effects) where agency is assumed to compensate for the lack of SES resources; or c) if there are cumulative effects, i.e. higher levels of agency are more beneficial at higher levels of parental SES.

### Structural Influences on Agency—a Multidimensional Conceptualization of SES

There is persistent evidence that a lack of family socioeconomic resources, i.e. poverty, loss of employment, or low levels of parental education, is associated with adjustment problems in offspring (Yoshikawa et al. 2012). For example, children born into less privileged families show, in general, lower levels of educational attainment (Bradley and Corwyn 2002; Engle and Black 2008; Schoon et al. 2002), educational achievement motivation (Duckworth and Schoon 2012; Mortimer et al. 2014; Schoon 2014), self-confidence and locus of control (Ahlin and Antunes 2015; Battle and Rotter 1963; Flouri 2006; Moilanen and Shen 2014). Explanations of these associations refer to cumulative risk effects (DiPrete and Eirich 2006), the lack of financial resources and socialization effects involving familiarity with the dominant culture, social networks and access to warm and supportive parenting (Conger et al. 2010).

Although the role of SES in shaping individual lives (including variations in locus of control) has been extensively studied, SES is treated in a variety of ways and there is still little understanding of the relative and independent role of multiple indicators of SES in shaping locus of control and experiences in the school-to-work transition. Moving beyond unidimensional conceptualizations of SES, such as a sole focus on parental education or income, or the use of a summary index comprising multiple indicators, we adopt a multidimensional approach in defining SES, “decomposing” the influence of socioeconomic risk (Bukodi and Goldthorpe 2013). In doing so, we assess the relative and independent contributions of multiple indicators of SES in shaping internal locus of control and transition experiences. In our analysis, we include indicators of longstanding financial advantage via assets such as home ownership, as well as more volatile or changeable indicators of financial status, i.e., current income levels and unemployment. These are assessed alongside traditional indicators of SES such as parental education and occupational class to provide broad coverage of parental SES.

Adopting a multidimensional definition of SES enables us to gain a better understanding of how internal locus of control might be shaped by different facets of social background—i.e., we can assess which dimensions of SES affect agency most strongly, and we gain a better understanding of whether internal locus of control can compensate for specific SES dimensions but not for others. Past research has shown that parental class, income, assets and education—relating to different forms of parental resources, such as economic, socio-cultural and informational—have independent and distinct effects on individual lives (Bukodi and Goldthorpe 2013; Duckworth and Schoon 2012). For example, youth living in families who own their own home might develop higher levels of internal locus of control because their lives are more predictable and stable (Saunders 1990). Better educated parents might help their children to develop skills and strategies to deal with problems effectively and thus raise their perceptions of control, self-reliance and personal responsibility (Ross and Mirowsky 2013), whereas working class parents with little control over their jobs might emphasize obedience and conformity (Bornstein and Bradley 2012; Lewis et al. 1999). Teens are likely to be aware of the vulnerabilities involved by living in poverty and in low income families, and the stress of this experience might undermine not only their parents’ capability for effective parenting but also their sense of personal control (Conger et al. 2010).

Understanding how SES affects developmental outcomes requires a careful differentiation of its elements, examining distinctive connections and influences. Likewise, a better understanding of how structural constraints and individual agency shape each other and how they interact during the transition from school to work requires the careful unpacking of the complex and potentially reciprocal relationships between different indicators of family background and individual characteristics and how these develop over time. To our knowledge this is the first study to assess the relative and independent role of multiple indicators of SES in shaping locus of control and their multivariate effect in shaping experiences in the school-to-work transition.

### Can Locus of Control Compensate for Socioeconomic Disadvantage?

Previous research has shown, that youth from less privileged families tend to leave education earlier and are more likely to encounter problems in the transition from school to work, such as experiencing prolonged periods of time spent not being in education, employment or training (NEET) than their more privileged peers (Bynner and Parsons 2002; Schoon and Lyons-Amos 2017, 2016). Longer periods of unemployment or economic inactivity during the school-to-work transition are problematic because this can increase the risk of poor occupational and psychological outcomes in the immediate and longer term including lower earnings, persistent unemployment, lower life satisfaction and higher levels of malaise (Bynner and Parsons 2002; Krahn and Chow 2016; Mroz and Savage 2006).

The transition from school-to-work is therefore likely to present additional difficulties for already vulnerable youth, magnifying prior risks as reflected in the notion of cumulative disadvantage (DiPrete and Eirich 2006)—or increasing opportunities for already privileged youth, reflecting cumulative advantage. However, each transition can demarcate a turning point that is associated with change for the better or worse (Elder and Shanahan 2006). There is heterogeneity in transition experiences and one of the most compelling reasons for longitudinal studies of youth transitions is to identify why some youth succeed against the odds, why some avoid negative outcomes such as long-term unemployment despite exposure to significant risk factors. We thus ask, if internal locus of control is a potential resource factor that can compensate for background disadvantage and enable youth to succeed against the odds.

The transition to independent adulthood is an important period for identity formation, where transition experiences may shape and have enduring consequences for youth’s self-concept and perception of control. The increasingly unstructured and protracted nature of the early adulthood period places an increased emphasis on youth finding and pursuing their own pathway to adulthood (Schwartz et al. 2005). In this respect, internal locus of control may be a key resource factor enabling the young person to navigate and take advantage of the available opportunities, while those who lack both structural support and agentic resources might more likely adopt “passive or procrastinatory approaches” placing them at risk of extended periods of not being in work, training or education (Schwartz et al. 2005).

Based on previous studies testing the compensatory role of personality characteristics and agency on status attainment outcomes after leaving compulsory schooling (Damian et al. 2015; Shanahan et al. 2014; Schoon and Lyons-Amos 2017), we test three patterns in relation to parental SES when predicting transition experiences: (1) the independent effects model, which assumes that parental SES and internal locus of control independently predict transition experiences; (2) the resource substitution, or compensatory model, which assumes interactions between SES and internal locus of control in that high levels of internal locus of control have more beneficial effects for youth from less privileged backgrounds; and (3) the cumulative advantage model, which expects that internal locus of control is a stronger predictor of attainment at higher levels of parental SES.

#### Independent effects

The independent effects model assumes no interactions between parental SES and internal locus of control, i.e., internal locus of control is beneficial for all youth across parental SES levels. Damian et al. (2015) refer to this as the default model, reflecting the standard approach taken by researchers assessing the validity of predictors, such as internal locus of control on outcomes such as academic attainment. For example, previous studies have shown that individuals who feel that they are in control do better academically (Au 2015; Bursik and Martin 2006; Lynch et al. 2002). There is however less current evidence on the role of internal locus of control as a predictor of experiences in the transition from school to work, nor have these prior studies tested for interactions between parental SES and internal locus of control in shaping subsequent transition experiences.

#### Compensatory effects

Compensatory effects are indicative of statistical moderation, i.e., distinct characteristics can moderate the disadvantaging effect of low socioeconomic resources. In other words, high levels of internal locus of control may compensate for background disadvantage (resource substitution) with respect to avoiding a problematic entry into the labor market, characterized by prolonged periods of NEET. If so, the benefits of high levels of locus of control would be greater for youth from disadvantaged backgrounds, enabling them to avoid a problematic transition. Preliminary support for this assumption was found in a cross-sectional study of 326 Greek undergraduate students, where internal locus of control seemed to buffer the negative effects of adversity measured using a life events checklist (Leontopoulou 2006). Compensatory effects regarding status attainment among less privileged individuals also have been identified related to personality characteristics (Shanahan et al. 2014). However, the compensatory effects were only weak and importantly they attenuated after controlling for indicators of cognitive ability—pointing to independent rather than compensatory effects (Damian et al. 2015). Thus, more research is needed to clarify the potential compensatory role of control perceptions in conditions of socioeconomic disadvantage.

#### Cumulative advantage

The first two models presume that human agency can to some extent enable individuals to steer the course of their lives, either independently of social background or by substituting for the lack of socioeconomic resources. The cumulative advantage model in contrast assumes that a favorable relative position produces further gains in developmental outcomes. This process has also been called a “Matthew effect”, suggesting that the rich get richer and that youth raised in relative privileged families are likely to benefit more from certain individual characteristics, probably because a more favorable environment enables the development and realization of relevant competences (Damian et al. 2015). According to the cumulative advantage model, we would expect youth from relatively advantaged backgrounds to have higher levels of internal locus of control (see Ahlin and Antunes 2015; Battle and Rotter 1963; Moilanen and Shen 2014) and to benefit more from this characteristic in the transition from school to work.

## Current Study

The aim of this study is to examine how youth’s internal locus of control and parental SES interact in shaping the transition from secondary school to work, focusing on the experience of prolonged NEET. Specifically, we test for evidence of four models: (1) the socialization effects model (i.e., youth with fewer parental socioeconomic resources are expected to have lower levels of internal locus of control); (2) the independent effects model (i.e., internal locus of control predicts time spent NEET independent of parental SES); (3) the resource substitution model (i.e., a high level of internal locus of control reduces the risk of being NEET especially at lower levels of parental SES); and (4) cumulative advantage model (i.e., a high level of internal locus of control reduces the risk of being NEET especially at high levels of parental SES).

We include prior academic attainment, gender and ethnic minority status as control variables to our model to take into account potential confounding factors. For example, previous studies have shown that academic attainment can increase feelings of being in control (Bandura 1997; Ross and Mirowsky 2013) and reduce the risk of being NEET (Bynner and Parsons 2002; Schoon and Lyons-Amos 2017). Regarding gender differences in internal locus of control, the evidence is not conclusive, with some studies finding that females have lower levels of control perceptions than males (see for example, Falci 2011) while others do not find significant differences (Ahlin and Antunes 2015; Moilanen and Shen 2014). Likewise, regarding the experience of NEET, some studies suggest that females have a higher risk of being NEET (Bynner and Parsons 2002), while others (using more current cohort data) find no significant differences (Duckworth and Schoon 2012; Schoon and Lyons-Amos 2017). Similarly, the evidence is not conclusive in relation to ethnic minority differences, with some studies in the US context suggesting that African Americans are less likely to develop high levels of internal locus of control than Whites (Ahlin and Antunes 2015), while others show that African Americans report higher levels of perceived control (Lewis et al. 1999) or find no significant differences (Moilanen and Shen 2014).

## Method

### Procedure and Sample

This study used data from the Longitudinal Study of Young People in England (LSYPE) which is a panel study of 15,770 youth born between 1st September 1989 and 31st August 1990. Sample members were youth in school year 9 (age 13/14) or equivalent, in England in February 2004. Annual face-to-face interviews were conducted with youth and their parents between 2004 and 2010 and data are linked to academic records from the National Pupil Data base (NPD) (for more details see https://www.education.gov.uk/ilsype/workspaces/public/wiki/Welcome).

The seven waves of data collection cover ages 13/14 (age at assessment: *M* = 14.26, SD = 0.32) to 19/20 (age at assessment: *M* = 20.31, SD = 0.31) years. The weighted sample is ethnically diverse though most minority ethnic groups are small, 86.1% identified as White, 2.5% Indian, 2.3% Pakistani, 0.9% Bangladeshi, 1.4% Black Caribbean, 1.6% Black African, 2.8% mixed ethnicity and 2.3% as other ethnicities. The sample is also diverse in terms of socioeconomic coverage, for instance, regarding maternal education, 11.3% of mothers had a degree or equivalent qualification, 12.6% had higher education below degree level, 13.5% had post-school qualifications (A-levels or equivalents), 30.3% had a good standard of secondary education (GCSEs at grade A–C), 9.9% had basic qualifications (level 1), 20.7% had no qualifications and 1.8% had other qualifications.

The LSYPE was sampled using a probability proportional to size method, using schools as the primary sampling unit. It was additionally stratified on deprivation levels of those schools, oversampling more deprived schools and pupils from minority ethnic groups. The initial sample size was 15,770 partial responses (data from young person) and 13,914 full responses (young person and parent) although not all youth provided information for all waves of the survey. The Wave 7 sample consisted of all youth who had been interviewed at previous waves and who agreed to be re-contacted. In total 9791 cases were contacted at Wave 7 in 2010.

### Measures

#### Internal locus of control

Internal locus of control was measured at wave 2, using a 3-item measure: If someone is not a success in life, it is usually their own fault (L1); I can pretty much decide what will happen in my life (L2); If you work hard at something you’ll usually succeed (L3). Responses were coded on 4-point scale 1 = strongly agree, 2 = agree, 3 = disagree, 4 = strongly disagree. Responses to all three items were reversed so high scores indicate higher levels of internal locus of control and low levels indicate low internal locus of control. Confirmatory factor analysis showed that the three items all loaded satisfactorily on a single factor (all std. loadings > .3 [L1: .34; L2: .36; L3: .50], *p* < .001).

#### Parental socioeconomic status (SES)

At wave 1 five indicators of parental SES were measured by parent-reported indicators of parental education, occupational class, income, housing tenure, and unemployment.

##### Parental education

was measured as the highest level of education of either parent on a 4-point scale, ranging from no or very low educational qualifications up to degree level qualifications.

##### Parental

household income was reported by the parent as annual banded income and was measured in four groups (i.e., <£10,400; £10,400-£20,800, £20,801-£33,800, >£33,800).

##### Parental occupational class

was assessed using the National Statistics Socioeconomic Classification, differentiating parents in routine and manual (1), intermediate (2), and higher professional and managerial (3) occupations—using the highest level of either parent.

##### Housing

was measured by parent reports, differentiating between home ownership (including mortgage, part-ownership, owned outright) and renting (including government-based and private renting).

##### Parental long-term unemployment

was identified if at least one parent was reported as being unemployed for over 6 months.

#### Time spent Not in Education, Employment or Training (NEET)

This was assessed with monthly activity history data collected over 44 months between ages 16 to 20 (October 2006 and May 2010). These data recorded youth’s main activity for each month during the year before the survey, including being in full-time education, employed (part- or full-time), in an apprenticeship or government training, or being NEET. For the current study, we created a variable which summed the number of occasions (i.e., months) youth reported being NEET.

#### Covariates

##### Academic attainment

was assessed by grades in Maths, English and Science (Key Stage 3) for Year 9 when pupils were approximately 13/14 years old, derived from the National Pupil Database.

##### Sex

Males were the reference category (coded 0) and females were the comparison group (coded 1).

##### Ethnicity

Due to the large number of relatively small minority groups in the UK we only controlled for ethnic minority status (coded 1) and compared this group to those of the majority white ethnicity group (coded 0).

### Statistical Analysis

Analyses consisted of a series of path models run using Mplus version 7 (Muthén and Muthén 2012). Due to the complex sampling strategy of the LSYPE, we utilized the cluster, stratification, and design weight options in Mplus. Similar to most longitudinal cohort studies the sample size reduced over time. Attrition was slightly higher among youth from lower SES backgrounds and lower academic attainment, but associations between observed characteristics and non-response were generally small (further details are available from the first author). The total available sample sizes were 15,770 at wave 1, 13,539 at wave 2, and 8682 at the wave 7 follow-up. Full activity data used to calculate time spent NEET were available for 8452 youth and partial responses were available for a further 3287. To reduce the bias arising from attrition, missing data were handled with full information maximum likelihood (FIML) which uses all available data (up to *N* = 15,770) rather than deleting participants or imputing values (Enders 2010).

## Results

Descriptive statistics for the main study variables are shown in Table 1. To simplify the table, dummy indicators for socioeconomic disadvantage were entered into the correlation matrix, rather than including dummies for all SES indicators (the dummy variables contrast indicators of low level SES to all other levels of SES as the reference group). All indicators of socioeconomic disadvantage were positively associated with months spent NEET while internal locus of control showed a negative correlation with months spent NEET. The control variables are also negatively associated with months spent NEET, suggesting less experience of time spent NEET among ethnic minority youth, females and those with higher academic attainment.

**Table 1:** Descriptive statistics for main study variables

		*M*	SD	1.	2.	3.	4.	5.	6.	7.	8.	9.
1.	iLoC (latent variable)	0.00	0.25									
2.	NEET	4.59	8.13	−.09^***^								
3.	Low occ. class	0.40	0.49	.00	.20^***^							
4.	No/low qualifications	0.22	0.41	.04^*^	.20^***^	.32^***^						
5.	Low income	0.17	0.38	.01	.15^***^	.19^***^	.24^***^					
6.	Rented tenure	0.27	0.44	.01	.27^***^	.34^***^	.33^***^	.32^***^				
7.	Long term unemployed	0.02	0.14	.00	.06^***^	.09^***^	.09^***^	.12^***^	.14^***^			
8.	Ethnic minority	0.12	0.33	.12^***^	−.03^**^	.02^**^	.18^***^	.10^***^	.08^***^	.09^***^		
9.	Female	0.48	0.50	−.05^*^	−.04^***^	−.01	−.01	.00	.00	.02^*^	.01	
10.	Academic attainment	0.08	0.99	.05^*^	−.36^***^	−.34^***^	−.32^***^	−.19^***^	−.32^***^	−.09^***^	−.06^***^	.05^***^

*Note*: Columns “1.” to “9.” show correlations

*N* = 15770. Full information maximum likelihood estimates

*iLoC*   internal locus of control, *NEET*  not in education, employment or training, *Low occ. class *  low occupational class comprising routine and manual occupations

^*^ *p* < .05, ^**^ *p* < .01, ^***^ *p* < .001

### Associations between Internal Locus of Control and SES

In contrast to our predictions of corrosive socialization effects (model 1), there was little evidence that SES was negatively associated with youth’s internal locus of control (see Table 2). Estimates were generally close to zero, however, in contrast to expectations, there was a slight tendency for youth from the richest group of households to report lower levels of internal locus of control. We also found small correlations with control variables, where higher internal locus of control was found for minority ethnic status, boys, and higher school attainment (see Table 1).

**Table 2:** Unadjusted associations for each socio-economic variable with internal locus of control

	B[95%CI]	*β*	R^2^
Occupational class			.00
Lower	.00 [−.02, .03]	.01	
Intermediate	(Ref)	(Ref)	
Higher	.00 [−.02, .03]	.01	
Education			.00
None/Low	.02 [−.01, .04]	.03	
Secondary	(Ref)	(Ref)	
FE	−.01 [−.03, .02]	−.01	
HE	−.01 [−.03, .02]	−.01	
Income			.00
<£10,400	−.01 [−04, .03]	−.01	
£10,400–£20,800	−.02 [−.04, .01]	−.03	
£20,801–£33,800	(Ref)	(Ref)	
>£33,800	−.03 [−.05, −.00]	−.05^*^	
Housing			.00
Owned	(Ref)	(Ref)	
Rented	.01 [−.01, .03]	.01	
Long-term unemployed			.00
No	(Ref)	(Ref)	
Yes	.00 [−.05, .06]	.00	

*Note*: The associations in this table are from separate path models for each socio-economic variable

*N* = 14997 (occupational class), 15532 (education), 14850 (income), 15698 (housing), 15665 (unemployment)

Full information maximum likelihood estimates

Data are weighted to population characteristics

^*^ *p* < .05

### Interactions of Internal Locus of Control and Socioeconomic Factors in Predicting Time Spent NEET

Bivariate associations presented in Table 3 show that socio-economic disadvantage is associated with more time being NEET. For example, living in rented housing was associated with an additional 4.86 months NEET compared to living in an owner-occupied home. As expected, internal locus of control was negatively associated with number of months spent NEET showing that higher internal locus of control was, on average, associated with fewer months NEET. In the multivariate model (Table 3), low parental occupational class and education, rented housing and internal locus of control all predicted duration NEET. Household income and parental unemployment did not. Moreover, internal locus of control has an independent effect on time spent NEET in addition to and above the influence of parental SES and the controls (gender, ethnicity, academic attainment). The findings thus support model 2. There was, however, some evidence of suppression effects in the multivariate model (higher occupational class predicted more time NEET) that were driven by inclusion of multiple (correlated) socioeconomic factors in the same model. Re-running the model for each socioeconomic factor separately did not result in suppression effects (results available from first author) so caution should be taken when interpreting the positive association between higher occupational class and time NEET.

**Table 3:** Main effects of socioeconomic factors and internal locus of control on time spent not in education, employment or training (NEET)

	Bivariate associations			Multivariate model + controls^a^		
	B[95%CI]	*Β*	R^2^	B[95%CI]	*β*	R^2^
Occupational class			.03			
Lower	2.64 [2.00, 3.27]	.16^***^		1.01 [0.40, 1.62]	.06^**^	
Intermediate	(Ref)	(Ref)		(Ref)	(Ref)	
Higher	−0.57 [−1.07, −0.08]	−.04^*^		0.68 [0.15, 1.20]	.04^*^	
Education			.04			
None/Low	2.83 [2.06, 3.60]	.15^***^		1.18 [0.46, 1.90]	.06^**^	
Secondary	(Ref)	(Ref)		(Ref)	(Ref)	
FE	−0.86 [−1.44, −0.29]	−.04^**^		−0.21 [−0.76, 0.33]	−.01	
HE	−1.63 [−2.08, −1.17]	−.09^***^		0.36 [−0.13, 0.85]	.02	
Income			.03			
<£10,400	2.74 [1.87, 3.60]	.13^***^		0.69 [−0.08, 1.45]	.03	
£10,400–£20,800	1.06 [0.45, 1.66]	.06^**^		−0.11 [−0.66, 0.45]	−.01	
£20,800–£33,800	(Ref)	(Ref)		(Ref)	(Ref)	
>£33,800	−1.42 [−1.90, −0.95]	−.08^***^		−0.37 [−0.85, 0.11]	−.02	
Housing			.07			
Owned	(Ref)	(Ref)		(Ref)	(Ref)	
Rented	4.86 [4.20, 5.51]	.27^***^		2.62 [1.99, 3.26]	.14^***^	
Long-term unemployed			.00			
No	(Ref)	(Ref)		(Ref)	(Ref)	
Yes	3.23 [1.26, 5.20]	.06^**^		0.50 [−1.35, 2.36]	.01	
Internal locus of control	−3.18 [−4.90, −1.46]	−.10^***^		−2.64 [−4.23, −1.06]	−.08^***^	
						.19

*Note*: Bivariate associations come from separate path models for each predictor variable

The multivariate path model contains all listed predictor variables and control variables

These models provide linear regression estimates using the Maximum Likelihood estimator

N for bivariate models = 14013 (occupational class), 15263 (education), 13519 (income), 15643 (housing), 15578 (unemployed). N for multivariate model = 15770

Full information maximum likelihood estimates

Data are weighted to population characteristics

^a^ Control variables were sex, ethnicity, and academic attainment

^*^ *p* < .05, ^**^ *p* < .01, ^***^ *p* < .001

Given the suppression effects found above, we examined interaction terms separately for each socioeconomic indicator. In line with the assumption of “resource substitution” (model 3), there were significant interaction effects suggesting that internal locus of control was particularly important to youth from low socioeconomic groups (Table 4). This applies to all five indicators of parental SES. The compensatory effects varied by the degree of disadvantage, where high levels of internal locus of control reduced time spent NEET for low socioeconomic groups but generally had little effect in the middle and especially high socioeconomic groups. We find no evidence to support model 4 regarding cumulative advantage. Indeed, the findings suggests that high levels of internal control among youth with higher educated parents are associated with an increased number of months spent NEET.

**Table 4:** Main and interaction effects of internal locus of control and socio economic factors on time spent not in education, employment or training (NEET). Unstandardised linear regression estimates and 95% confidence intervals

	Model i.		Model ii.		Model iii.		Model iv.		Model v.	
	Main effect	Interaction	Main effect	Interaction	Main effect	Interaction	Main effect	Interaction	Main effect	Interaction
	B [95%CI]	B [95%CI]	B [95%CI]	B [95%CI]	B [95%CI]	B [95%CI]	B [95%CI]	B [95%CI]	B [95%CI]	B [95% CI]
Occupational class										
Lower	**2.01** [1.22, 2.80]	**−5.51** [−7.14, −3.88]								
Intermediate	(Ref)	(Ref)								
Higher	0.20 [−0.32, 0.71]	1.12 [−0.41, 2.66]								
Education										
None/Low			**2.98** [1.86, 4.09]	**−4.59** [−6.63, −2.56]						
Secondary			(Ref)	(Ref)						
FE			−0.56 [−1.19, 0.08]	**2.96** [0.73, 5.20]						
HE			−0.42 [−1.05, 0.21]	**3.57** [1.18, 5.95]						
Income										
<£10,400					**2.19** [1.10, 3.28]	**−5.76** [−7.92, −3.60]				
£10,401–£20,800					**0.61** [0.02, 1.20]	1.27 [−4.43, 6.96]				
£20,800–£33,800					(Ref)	(Ref)				
>£33,800					**−0.49** [−0.97, −0.01]	0.82 [−0.17, 1.81]				
Housing										
Owned							(Ref)	(Ref)		
Rented							**3.79** [2.85, 4.73]	**−8.95** [−9.93, −7.97]		
Long-term unemployed										
No									(ref)	(ref)
Yes									1.97 [−0.32, 4.25]	**−6.24** [−10.58, −1.90]
Internal locus of control	−1.32 [−2.82, 0.18]		**−3.61** [−6.01, −1.20]		−0.84 [−1.79, 0.10]		**−0.39** [−0.71, −0.08]		**−0.53** [−0.86, −0.20]	
Ethnic minority	0.05 [−0.48, 0.58]		−0.28 [−0.82, 0.25]		−1.01 [−2.10, 0.09]		−0.33 [−0.73, 0.07]		**−0.94** [−1.36, −0.52]	
Female sex	**−0.73** [−1.10, −0.36]		**−0.70** [−1.07, −0.32]		**−0.47** [−0.90, −0.03]		**−0.67** [−1.01, −0.33]		**−0.46** [−0.82, −0.09]	
Academic attainment	**−1.94** [−2.35, −1.53]		**−2.05** [−2.45, −1.65]		**−2.43** [−2.78, −2.08]		**−2.04** [−2.30, −1.77]		**−2.77** [−3.04, −2.50]	
Wald test for interactions		*P* < .0001		*P* < .0001		*P* < .0001		*P* < .0001		*P* < .01

*Note*: *N* = 12802, 14964, 11427, 15582, 15489 for models i–v, respectively

Full information maximum likelihood estimates

Data are weighted to population characteristics

Significant estimates (*p* < .05) are given in bold

As a robustness check, we used a dichotomous measure of time spent NEET, differentiating between a prolonged period of being NEET (6 months or longer) which affected about 20% of the sample and those with fewer months being NEET. When using the dichotomous outcome measure in a logistic regression model, the interaction effects between high levels of internal control and the indicators of parental SES ceased to be significant (Table 5). Internal locus of control continued to show a significant independent effect over and above indicators of parental SES.

**Table 5:** Main and interaction effects of internal locus of control and socio economic factors on prolonged time (6 months and more) spent not in education, employment or training (NEET). Logistic Regression Results

	Model i.		Model ii.		Model iii.		Model iv.		Model v.	
	Main effect	Interaction	Main effect	Interaction	Main effect	Interaction	Main effect	Interaction	Main effect	Interaction
	Odds Ratio [95%CI]	Odds Ratio [95%CI]	Odds Ratio [95%CI]	Odds Ratio [95%CI]	Odds Ratio [95%CI]	Odds Ratio [95%CI]	Odds Ratio [95%CI]	Odds Ratio [95%CI]	Odds Ratio [95%CI]	Odds Ratio [95% CI]
Occupational class										
Lower	**1.49** [1.23, 1.80]	1.18 [0.85, 1.63]								
Intermediate	(Ref)	(Ref)								
Higher	1.13 [0.93, 1.38]	1.17 [0.83, 1.65]								
Education										
None/Low			**1.45** [1.20, 1.75]	1.01 [0.74, 1.36]						
Secondary			(Ref)	(Ref)						
FE			0.86 [0.70, 1.05]	1.23 [0.87, 1.73]						
HE			1.01 [0.85, 1.20]	1.25 [0.92, 1.70]						
Income										
<£10,400					**1.46** [1.16, 1.84]	0.86 [0.59, 1.26]				
£10,401–£20,800					**1.24** [1.04, 1.49]	1.25 [0.89, 1.76]				
£20,800–£33,800					(Ref)	(Ref)				
>£33,800					0.81 [0.66, 1.01]	1.18 [0.81, 1.71]				
Housing										
Owned							(Ref)	(Ref)		
Rented							**1.94** [1.68, 2.23]	0.87 [0.68, 1.10]		
Long-term unemployed										
No									(Ref)	(Ref)
Yes									1.39 [0.90, 2.16]	0.73 [0.31, 1.72]
Internal locus of control	**0.74** [0.56, 0.99]		**0.73** [0.58, 0.91]		**0.77** [0.60, 0.99]		**0.85** [0.74, 0.96]		**0.82** [0.73, 0.91]	
Ethnic minority	**0.75** [0.63, 0.91]		**0.68** [0.58, 0.81]		**0.67** [0.55, 0.82]		**0.71** [0.61, 0.83]		**0.74** [0.63, 0.87]	
Female sex	**0.77** [0.68, 0.88]		**0.79** [0.70, 0.89]		**0.80** [0.69, 0.92]		**0.79** [0.70, 0.89]		**0.81** [0.72, 0.91]	
Academic attainment	**0.49** [0.45, 0.53]		**0.49** [0.46, 0.53]		**0.51** [0.47, 0.56]		**0.51** [0.47, 0.55]		**0.47** [0.44, 0.51]	
Wald test for interactions		*P* = .59		*P* = .34		*P* = .14		*P* = .49		*P* = .47

*Note*: *N* = 12802, 14964, 11427, 15582, 15489 for models i–v, respectively

Full information maximum likelihood estimates

Data are weighted to population characteristics

Significant coefficients (*p* < .05) are given in bold

## Discussion

This article examined a number of hypotheses about the inter-relations between parental SES and youth’s internal locus of control during the transition out of secondary school education. We found no support for our first model which predicted associations between low parental SES and lower levels of internal locus of control in youth (Table 2). This is somewhat surprising given that earlier studies have quite consistently reported higher internal locus of control among children of middle class parents (Ahlin and Antunes 2015; Battle and Rotter 1963; Flouri 2006; Moilanen and Shen 2014). However, our null findings complement recent work showing that among current cohorts control perceptions and belief in upward mobility is high among the majority of youth regardless of parental SES (Shane and Heckhausen 2017). Therefore, one interpretation of our findings might be that youth in the UK today are relatively optimistic of their own sense of control regardless of their parents’ socioeconomic position. This may be true given that the UK had a broadly comprehensive school system and widespread access to higher education during the period these youth were studied, and the majority of youth, including those from less privileged backgrounds aimed to participate in higher education (Schoon 2014). As such, it may be more fruitful to examine more proximal processes in children’s lives, such as interactions with parents, peers and teachers as potential drivers of individual differences in internal locus of control (see for example, Ahlin and Antunes 2015; Moilanen and Shen 2014). A second interpretation is that mid-adolescence is not the best time to measure social class given that much of the existing literature points to early childhood as an important period for the effects of social inequalities (Duncan et al. 2010; Heckman 2006). Future research might be able to tease these associations apart by following youth from an earlier age than we do here.

Greater understanding of how high internal locus of control can be cultivated is warranted if its efficacy as a protective factor is to be tested more fully. The associations found between internal locus of control and covariates suggest higher levels of internal locus of control among males, ethnic minorities and those doing better academically (Table 1). As such, increased personal agency may come about from greater understanding of structural constraints (e.g., as communicated in gender or racial/ethnic socialization processes) or from positive reinforcement (e.g., academic success). Parents are likely to play an important role in their children’s perceptions of control and the extant literature demonstrates some interesting differences in child-rearing between working- and middle-class parents (Bornstein and Bradley 2012; Ross and Mirowsky 2013) as well as gender differences in socialization. For instance, parents are more inclined to instill self-direction and initiative among their sons than among their daughters (Falci 2011). Moreover, middle-class parents tend to place greater value on self-direction that focuses on the child internally directing their own behavior, while working-class parents tend to place greater value on conformity which focuses on behavior controlled by externally imposed conditions (Kohn and Schooler 1983; Lewis et al. 1999). It is possible that the children from low SES families with high levels of internal locus of control are a slightly unusual group and this is possibly due to their experience of parenting that has traditionally been more common in middle-class families. Interestingly, we found a negative association between internal locus of control and high levels of parental income, potentially pointing to a more carefree environment among relatively privileged youth—however, the association was only very small.

Multiple dimensions of SES are associated with the duration youth spent NEET. The most robust of these negative effects relate to rented housing status, low parental occupational class and low parental education (Table 3). Parental housing status is by far the strongest of these predictors, approximately twice as strong as education or occupational class in fact. Home ownership is a key indicator of wealth and assets and its strong predictive effect suggests youth’s difficulties establishing productive roles after secondary school are driven by more pervasive and ingrained inequalities, the availability of assets and potentially also neighborhood characteristics (Ahlin and Antunes 2015; Schoon 2014; Schoon and Lyons-Amos 2017).

Additional variance is explained by internal locus of control over and above these SES effects, supporting model 2 regarding independent effects (Table 3). As expected, all indicators of SES predicted the experience of NEET. Yet, the findings also show that internal levels of control have significant main effects on the experience of NEET, even after controlling for SES, academic attainment, gender and ethnic minority status. The findings thus suggest that individuals are not passively exposed to structural forces and to some extent can steer the course of their life despite socioeconomic constraints. Perceptions of control are an important prerequisite for taking action in the face of difficulties or uncertainty and such perceptions have shown to play an important role in youth’s transitions to adulthood (e.g., Lewis et al. 1999). Interestingly, the findings in the multivariate model (Table 3) suggest that after controlling for the other indicators of SES, parental income and parental unemployment were not significant predictors of NEET, confirming previous evidence that challenges the assumption of an intergenerational transmission of a “culture of worklessness”, rather pointing to the role of cumulative risks in the lives of the most disadvantaged families (Schoon 2014).

Moreover, a pattern of statistical interactions across all measures of SES supported the assumption of “resource substitution” (model 3). The resource substitution model states that if a resource such as high SES or high internal locus of control is absent, it can be compensated by the other, with each having less of an effect if the other is present and the worse outcomes found for those with neither resource (Ross and Mirowsky 2006). Social privilege provides confidence in one’s worth, while perceptions of control provides confidence in one’s ability, and either of these resources serves as an alternative means of reducing the risks posed by the transition from school to work (Ross and Mirowsky 2013). The findings suggest that internal locus of control is an important predictor of the number of months spent NEET, especially for youth with the fewest socioeconomic resources.

However, when testing the robustness of these effects by using a dichotomized score differentiating between longer experiences of being NEET (6 months and longer) during the 4-year period after leaving compulsory schooling and less precarious transitions, we find that the significant interaction effects cease to be significant (Table 5). Thus, while internal locus of control might be advantageous in reducing the risk of occasional periods of economic inactivity or unemployment, it is less effective in protecting against the prolonged experience of being NEET among less privileged youth. We find independent effects for internal locus of control over and above parental SES in all the models predicting prolonged time spent NEET. The findings thus suggest, that transition experiences are especially challenging for youth who might not have access to the necessary information and guidance or financial resources. As already indicated above, a promising avenue for future studies would be to investigate family socialization practices in more detail, and how these are interlinked with different dimensions of parental SES.

Regarding model 4, we found little evidence to support the assumption of cumulative advantage. As already mentioned, we found no strong associations between parental SES and internal locus of control among youth—and interestingly, internal locus of control typically had very little influence on the time spent NEET among youth from middle and especially high SES backgrounds. In fact, youth with high levels of internal locus of control growing up with better educated parents showed an increased risk for experiencing NEET (although not prolonged NEET of 6 months or more). The findings thus point to a potential “dark side” of high control perceptions, which for some can imply that they overestimate their capability to find employment. This might be especially critical in times of an economic downturn, such as the Great Recession that occurred just at the time when this cohort of youth made their way into the labor market. For privileged youth more generally, the findings may reflect the reality that they do not perceive any structural constraints regarding their progression from school to future ventures. It might be that because the opportunities open to relatively privileged youth are largely structurally determined these youth require little agency to succeed or that they rely on their parents to support them.

In sum, the different effects seen for low vs. high SES youth are indicative of internal locus of control being a “resilience” factor rather than a universally “promotive” factor, where resilience is defined as a better outcome than is usually expected from individuals with a similarly adverse background (Rutter 2015). We do however note that the beneficial effects of internal locus of control for relatively disadvantaged youth are not unlimited, and that high levels of control perceptions do not enable disadvantaged youth to ward off prolonged experiences (6 months or more) of being NEET.

There are some limitations to the current study that future studies should aim to improve upon. For example, future research should aim to use improved measures of internal locus of control. The factor loadings for the scale used here, while within typically accepted criteria (Brown 2006), were low which is likely to have attenuated the reported effect sizes. The effects reported here should therefore be considered conservative with potential for much stronger effects (Frost and Thompson 2000). We used a brief (3-item) questionnaire scale as this was the measure available in the LSYPE, but future research should aim to use more reliable scales, such as the 20 item Nowicki-Strickland scale (1973). It would also be beneficial to measure parents’ locus of control in future studies to assess the extent to which control perceptions are transmitted between generations. Previous research found the compensatory effects of personality attenuated after controlling for intelligence (Damian et al. 2015). Measures of intelligence were not available in the LSYPE dataset, so we controlled for academic attainment instead. Intelligence and school achievement tend to be highly correlated—yet they are clearly distinct constructs with their own strengths and biases. Future studies should thus test, whether the findings presented in this article hold in a sample containing measures of intelligence. Moreover, as in all longitudinal studies we are faced with the problem of missingness in response. We used full information maximum likelihood estimates to address this issue. Moreover, we checked the robustness of findings against results with complete data, which confirmed the stability of the solution. Finally, our findings might be unique to the English context, especially regarding variations in experience among minority youth.

## Conclusion

The findings suggest that internal locus of control can to some extent protect disadvantaged youth from precarious transition experiences after the completion of compulsory education—however it does not protect against prolonged experiences of economic inactivity and unemployment during the post-school period. Thus, agency appears to be most effective when socioeconomic constraints are not overpowering. We showed that internal locus of control can in some circumstances compensate for background disadvantage, even after controlling for academic attainment. There are significant effects at both ends of the internal locus of control continuum, and variations of interactions for high and low SES groups. The findings highlight the importance of adopting a multi-dimensional conceptualization of SES, and considering interactions between individual agency and distinct dimensions of socioeconomic adversity to get a better understanding of developmental processes during important transition periods.
